# An Anion Conductance, the Essential Component of the Hydroxyl-Radical-Induced Ion Current in Plant Roots

**DOI:** 10.3390/ijms19030897

**Published:** 2018-03-18

**Authors:** Igor Pottosin, Isaac Zepeda-Jazo, Jayakumar Bose, Sergey Shabala

**Affiliations:** 1 Centro Universitario de Investigaciones Biomédicas, Universidad de Colima; Av. 25 de julio 965, Villa de San Sebastian, Colima, Col. 28045, Mexico; 2 School of Land and Food, University of Tasmania, Private Bag 54, Hobart, Tas. 7001, Australia; sergey.shabala@utas.edu.au; 3 Genómica Alimentaria, Universidad de La Ciénega del Estado de Michoacán de Ocampo, Av. Universidad 3000, Lomas de la Universidad, Sahuayo, Mich. 59103, Mexico; z_isaac@hotmail.com; 4 Australian Research Council Centre of Excellence in Plant Energy Biology, School of Agriculture, Food and Wine, University of Adelaide, Adelaide SA 5064, Australia; jayakumar.bose@adelaide.edu.au

**Keywords:** anion conductance, electrolyte leakage, hydroxyl radical, membrane potential, MIFE, oxidative stress, patch-clamp

## Abstract

Oxidative stress signaling is essential for plant adaptation to hostile environments. Previous studies revealed the essentiality of hydroxyl radicals (HO•)-induced activation of massive K^+^ efflux and a smaller Ca^2+^ influx as an important component of plant adaptation to a broad range of abiotic stresses. Such activation would modify membrane potential making it more negative. Contrary to these expectations, here, we provide experimental evidence that HO• induces a strong depolarization, from −130 to −70 mV, which could only be explained by a substantial HO•-induced efflux of intracellular anions. Application of Gd^3+^ and NPPB, non-specific blockers of cation and anion conductance, respectively, reduced HO•-induced ion fluxes instantaneously, implying a direct block of the dual conductance. The selectivity of an early instantaneous HO•-induced whole cell current fluctuated from more anionic to more cationic and vice versa, developing a higher cation selectivity at later times. The parallel electroneutral efflux of K^+^ and anions should underlie a substantial leak of the cellular electrolyte, which may affect the cell’s turgor and metabolic status. The physiological implications of these findings are discussed in the context of cell fate determination, and ROS and cytosolic K^+^ signaling.

## 1. Introduction

Oxidative stress signaling is a common component of plant adaptation to a hostile environment [1,2,3,4,5]. In the last two decades, it became well established that reactive oxygen species (ROS) induce non-selective cation currents across the plasma membrane [6,7]. This has two major consequences, namely increased Ca^2+^ influx and K^+^ loss. ROS-induced Ca^2+^ influx is essential for growth and development and responses to environmental clues; in case of the developmental and stress responses, ROS may evoke both survival and programmed death scenarios [4,5,8]. Local Ca^2+^ responses may propagate over a long distance; this Ca^2+^ wave is associated with, and supported by, an electropotential and/or ROS waves [9,10]. More recently, the role of ROS-induced K^+^ efflux was recognized. Stress-induced K^+^ loss could turn on the programmed cell death scenario or switch the cell metabolism to a hibernation mode, reducing the energetic burden (reviewed by [7,11,12]).

However, a substantial K^+^ loss is only possible in a virtually electroneutral mode. In other words, the cytosolic K^+^ leak needs to be balanced by an external cation influx or to be coupled with a co-transport of anions. Salt stress represents a unique situation when an external electrolyte is present at a high concentration. Even though, high salt-induced cation fluxes, namely Na^+^ influx, overwhelms K^+^ efflux, requiring a substantial Cl^−^ influx for the sake of the electroneutrality. With only a few exceptions, when salt-induced Cl^−^ influx was measured in parallel with K^+^ and Na^+^ fluxes [13], Cl^−^ fluxes are usually overseen. This is largely explained by methodological issues related to measurements of anion fluxes by means of self-reference ion-selective electrodes in vivo [14]. It should be also added that under low external salt conditions the role of anion efflux to electrically balance the stress-induced K^+^ efflux might become even more relevant.

Hydrogen peroxide (H_2_O_2_) and hydroxyl radicals (HO•) are two ROS species which directly activate ion currents across the plasma membrane in plants. It appears that H_2_O_2_-induced currents are restricted to the growing tips [15]. HO•-induced currents occur more universally but seem to be more heterogeneous. One of the HO•-induced currents is mediated by an outward-rectifying K^+^-selective channels, GORK [16]. Other HO•-induced currents display poor selectivity among mono- and divalent cations, mediating both K^+^ efflux and Ca^2+^ influx [17,18]. Even more surprisingly, early component of the HO•-induced current in the root plasma membrane, termed ROSIC, appears to conduct Cl^−^ and a variety of cations, both small (K^+^, Ca^2+^) and relatively large (TEA^+^), but excluding large anions like HEPES [19]. In the present study, we used pea and barley roots to present further evidence for a substantial anion component of the HO•-induced conductance in vivo. The unitary properties, selectivity and temporal dynamics of the HO•-induced current in the isolated root protoplasts are analysed and discussed in the context of plant adaptation to adverse environmental conditions.

## 2. Results

**Figure 1:**
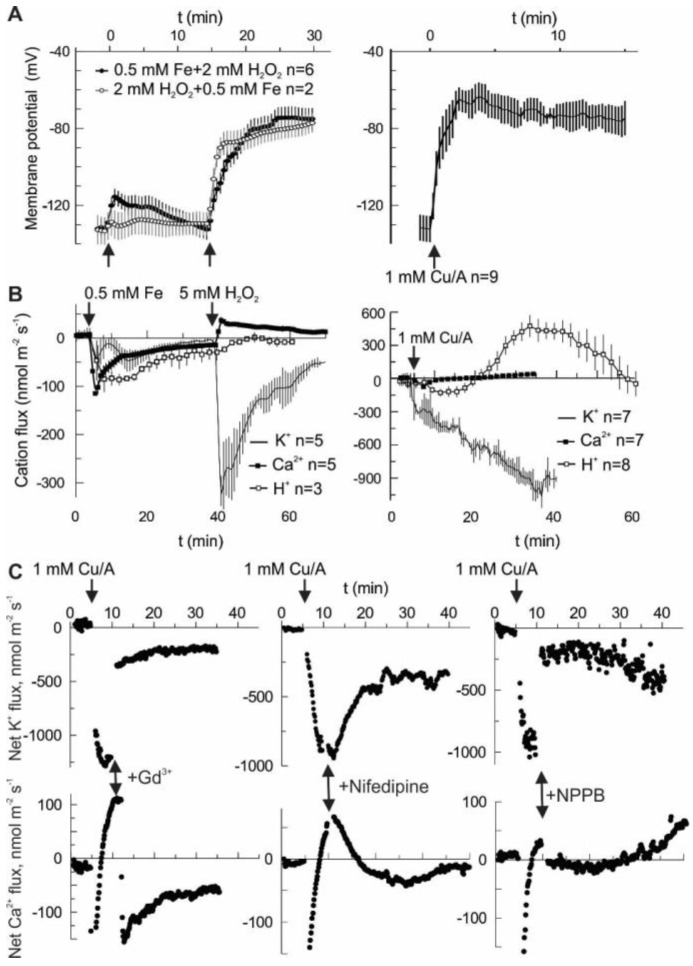
Evidence for the anionic component of HO•-induced ion flux in vivo. (**A**) HO• but not H_2_O_2_ provokes a substantial sustained depolarization in the mature zone of intact pea roots. HO• are generated either by mixing of copper with ascorbate in aerated BSM solution or by reducing of H_2_O_2_ by iron. (**B**) Cationic (Ca^2+^, K^+^ and H^+^) fluxes evoked by same treatments as in (**A**). The negative flux corresponds to *efflux* of cation. It can be seen that at all times there is net cation efflux, so that membrane depolarization requires even larger efflux of intracellular anions. (**C**) Passive cationic (Ca^2+^ and K^+^) fluxes are blocked not only by the direct application of cationic blockers (Gd^3+^ and nifedipine) but also by the anionic blocker NPPB. Blockers were applied at the moment, when passive Ca^2+^ influx clearly dominated over the Ca^2+^ pumping. Note the instantly developed block by Gd^3+^ and NPPB.

Hydroxyl radicals (HO•) may be generated by the Fenton reaction, using a copper-ascorbate (Cu/A) mixture or by combining iron ions with H_2_O_2_. In both cases, the treatment provoked a large and sustained depolarization in the pea root mature epidermis, from the resting value of −130 ± 5 mV to about −70 mV. Notably, H_2_O_2_ alone did not produce a significant change of membrane potential (Figure 1A). Depolarization may be produced by a net influx of cations and/or by efflux of the intracellular anions. However, an influx of cations is limited by their low concentration in the external medium, which contained 0.5 mM KCl and 0.1 mM CaCl_2_ under our experimental conditions. Figure 1B shows that in the first 30 min after HO• stimulation (second arrow in Fe-H_2_O_2_ treatment) the net cation flux was dominated by K^+^ efflux, whereas Ca^2+^ influx was small at any time. The same is true for the Cu/A treatment. In the latter case, H^+^ flux followed a complex kinetics. Initially, H^+^ pumping is started, most likely to counteract membrane depolarization. Later on, the net H^+^ influx is observed, possibly as a result of activation of a H^+^ co-transport (e.g., K^+^/H^+^ symport) mechanism. Whatever is the cause, in the first 30 min the net flux of K^+^, H^+^ and Ca^2+^ will tend to hyperpolarize the membrane potential and may not explain the observed fast depolarization (Figure 1A). Previously, we have reported the effects of different blockers on the HO•-induced K^+^ and Ca^2+^ fluxes and whole-cell currents [19]. In this study, we are presenting the effects of acute application of three selected blockers. This includes Gd^3+^, non-specific blocker of non-selective cation channels (NSCC); nifedipine, a blocker of L-type Ca^2+^ channels in animals and ROS-activated currents in plants; and NPPB, a non-specific blocker of anion-selective pores [20,21]. Both HO•-induced K^+^ efflux and Ca^2+^ influx were blocked by Gd^3+^ and NPPB almost instantaneously, whereas the onset of the block by the lipophilic nifedipine was relatively slow (Figure 1C). NSCC in plants normally conduct di- and monovalent cations with a little preference [21], so the simultaneous effect of Gd^3+^ on HO• -induced K^+^ and Ca^2+^ fluxes may be easy to understand if these fluxes are mediated by a NSCC. Yet, the effect of NPPB implies that the HO•-induced conductance contains a substantial anion component. Moreover, by blocking this component, K^+^ and Ca^2+^ fluxes need to be reduced in a coupled manner. The simplest way to explain such a coupling would be a pore which conducts both cations and anions. Presumably, such a non-selective pore should be characterized by a relatively high unitary conductance.

**Figure 2:**
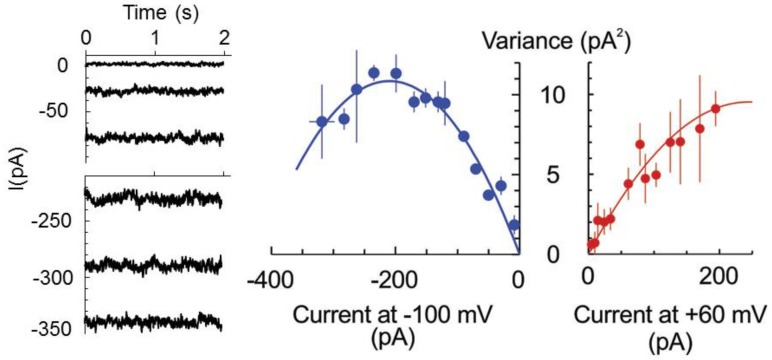
HO•-induced current is mediated by low conductance pores. Example of a steady-state HO•-induced whole-cell current at −100 mV, measured in the epidermal protoplast isolated from the mature zone of pea root. Measurements were taken at different time points after the initiation of treatment, reflecting the development of whole-cell current and respective noise pattern. The records are low-pass filtered at 2 KHz. Currents were averaged at fixed voltages of −100 and +60 mV, and the current variance (σ^2^) was calculated by subtracting this value from the actual current value and then raising the respective numbers to a second power. Mean σ^2^ values were plotted against the respective mean current values for three individual protoplasts, which displayed comparative magnitudes of limiting HO•-induced whole-cell currents (in the range of −300–−350 pA at −100 mV). Data are means ± SE. Solid lines are best fits to equation σ^2^ = *i*I − I^2^/N, with unitary current values, *i*, of −110 fA and +80 fA for −100 and +60 mV. See text for more details.

The latter hypothesis was verified by studying the whole cell currents by the patch-clamp technique. Previously, we have never observed discrete current steps of large (pA) amplitude upon the development of HO•-induced whole-cell current [10,19,22]. The possible (although unlikely) explanation for this fact may be that, once opened, the channel remained open all the time, i.e., open probability is equal to one, *P_0_* = 1. Alternatively and more likely, the HO•-activated pores are of the too small conductances so that single channel closed-open transitions could not be resolved. In this case, the non-stationary noise analysis of whole cell current can be applied to determine the number of open channels and unitary current magnitude [23]. Briefly, the magnitude of the noise (variance) depends on the open probability (hence, macroscopic current at a fixed potential) in a parabolic way: σ^2^= *i*I − I^2^/N, where *i* and I are the single channel and macroscopic current, respectively, and N is the number of channels. The maximal noise is observed when the channel open probability *P_0_* = 0.5 or I = N*i*/2. Assuming that the number of pores is fixed and the stimulus (here, HO•) only increased their open probability in time, we have fitted the data at two fixed potentials, −100 and +60 mV for three protoplasts, which displayed a similar magnitude of the limiting HO•-induced instantaneous current, reached at 30–40 min of exposition to copper/ascorbate mixture (Figure 2). For −100 mV, the maximum in the dependence of σ^2^ from the macroscopic current magnitude was apparent so that maximal open probability of 0.69 exceeded 0.5. For +60 mV, the maximal open probability was <0.5, implying a modest modulation of the open probability by the membrane voltage. The unitary currents of −110 and 80 fA were determined for −100 and +60 mV, respectively, which yields the unitary conductance of 1.1 pS.

**Figure 3:**
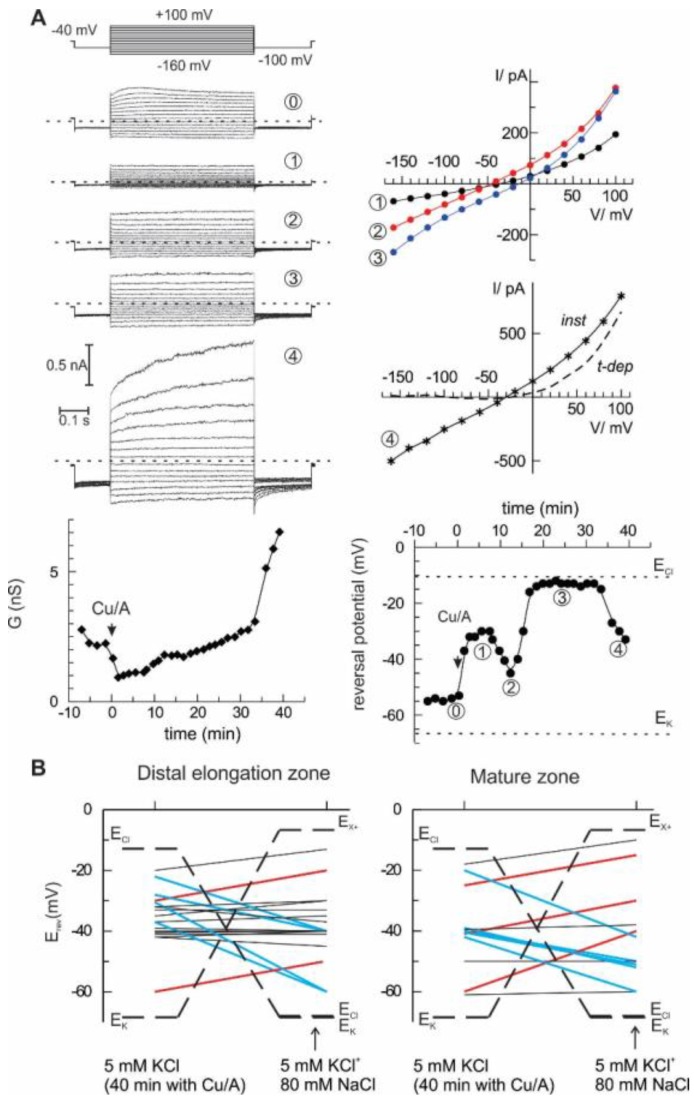
Ionic selectivity of the HO•-induced whole-cell current is variable. (**A**) An example of the development of HO•-induced current in isolated barley root epidermis protoplast from the elongation zone. The current responses to a sequence of voltage steps were recorded at different time points (examples are at the left-hand side; dotted lines indicate zero current). The current-voltage relationships for HO•-induced currents are plotted at the right (for the last record both instantaneous, *inst*, and time-dependent components, *t-dep*). Beneath are the time course for the changes in whole cell conductance and zero current potential; dotted lines are equilibrium potential values for K^+^ and Cl^−^. (**B**) A variability of the cation/anion selectivity of the HO•-induced whole-cell current between individual protoplasts. After 40 min of HO• treatment, the reversal potential of the whole cell current was evaluated, and then low salt bath was supplemented with 80 mM NaCl and reversal potential was evaluated again. E_K_ and E_Cl_ are Nernst potentials for K^+^ and Cl^−^, E_X_^+^ corresponds to a theoretical reversal potential for a hypothetical current, strictly selective for monovalent cations over anions, but which does not differentiate between K^+^ and Na^+^. The lines connect reversal potential values before and after addition of high-NaCl bath, red and blue ones are for preferential anionic and cationic selectivity, respectively.

The most direct way to quantify the selectivity of the ion current is to analyse its current-voltage relationship and to evaluate the zero-current (reversal) potential under voltage-clamp conditions and contrasting ionic gradients. To do this, epidermal protoplasts from barley root mature and distal elongation zones were used as a model system. Initially, the bath solution contained 6 mM K^+^ and 10 mM Cl^−^, whereas pipette solution had 104 mM K^+^ and 7.6 mM Cl^−^ (see Materials and Methods for detailed composition). Rectangular voltage steps ranging from −160 to +100 mV were applied, and steady-state currents were measured and plotted against the membrane potential, yielding current-voltage relationships. This procedure was repeated every 2 min, starting from control and continuing with the HO• treatment. Two major characteristics of the whole cell current were determined: a chord conductance and a reversal potential value (see example in Figure 3A). In this example, the reversal potential values fluctuated in time, indicating the changes between cationic or anionic preference (see for instance an abrupt change between points 2 and 3, which occurred without a large change in whole cell ion conductance). After 40 min of treatment with HO•, additional 80 mM NaCl was introduced into the bath, and the reversal potential of the whole cell current was measured again. The summary of these measurements is given in Figure 3B. Note that originally, equilibrium potential for K^+^ was much more negative than that for Cl^−^. After addition of 80 mM NaCl, E_Cl_^-^ approached E_K_^+^ (about −70 mV). At the same time, theoretical reversal potential for a current, which is strictly selective for monovalent cations against Cl^−^, but cannot differentiate between Na^+^ and K^+^, E_X_^+^, is much more positive. Obviously, the reversal potential for any passive ionic current has to be situated between these two limits. The upward deflection of the reversal potential value upon solution exchange implies a domination of the cation selectivity (red lines), whereas the domination of an anion (Cl^−^) selectivity over Na^+^ and K^+^ is manifested by a downward deflection (blue lines). In many cases, no significant change of reversal potential was observed (black lines), which either implies an approximate balance between cationic and anionic components, or, as in the two cases for protoplasts from the mature zone, the domination of a K^+^-selective current. In the previous study [10], we have applied the same procedure, but simply averaged whole-cell currents for all protoplasts before and after the addition of high NaCl into the bath. The result was predictable; no significant change of the reversal potential was detected as if the mean current was non-selective between monovalent cations and Cl^−^. A more detailed analysis of single-cell currents as in Figure 3 implies that the relative selectivity may be changing in time and varies between individual protoplasts. This is true for the instantaneous HO•-induced current developed at early times. At longer (more than 30 min) times of the treatment, a sudden increase of the whole cell was frequently observed, due to a development of the time-dependent component (Figure 3A, the last record). This time- and a voltage-dependent component likely reflects the activation of the outwardly rectifying K^+^-selective GORK channels, consistent with previous reports [16,24].

## 3. Discussion

This study emphasizes a significant contribution of the anion component in the flux response to extracellularly generated HO•. In vivo measurements demonstrate that the sum of cation fluxes may not explain the HO•-induced depolarization and implies a need for a substantial efflux of intracellular anions (Figure 1). Moreover, HO•-induced cation fluxes were blocked not only by the cation channel blocker Gd^3+^ but also by the anion conductance blocker, NPPB. This may be easily understood if one assumes a non-selective pore, where cation fluxes share the same conductance route as Cl^−^ and other small monovalent anions. This is not excluded by the present data, but seems less probable, due to a very low unitary conductance of HO•-activated early instantaneous currents (Figure 2) and variable (also, in time) contribution of the anionic and cationic current components (Figure 3). An alternative view is that HO• activates two types of conductance, specific for anions and cations. This suggestion may serve as an attractive explanation of the fluctuations in the relative selectivity of the HO•-activated whole-cell currents (Figure 3). One needs just to presume that the relative contribution of these two components with the contrasting selectivity may vary from protoplast to protoplast and is not fixed in time. Interestingly, either Gd^3+^ or NPPB produced the block of K^+^ efflux by 80% (Figure 1C). In variance to this, HO•-activated whole-cell current under voltage-clamp conditions was roughly halved by either of these compounds [19]. This may be due to the approximately equal contribution of anion and cation components, each of them being almost completely suppressed by Gd^3+^ and NPPB, respectively. However, when voltage is free running, as in vivo, due to the electrocoupling, a reduction of the K^+^ efflux will also reduce the efflux of intracellular anions (Cl^−^) and vice versa. Thus, under physiological conditions, the effect of either of blockers, cationic or anionic one, will be more pronounced as compared to voltage-clamp (patch-clamp) conditions when the electrocoupling restriction is lifted.

An important consequence of a parallel flow of K^+^ and intracellular anions is a possibility to cause a substantial reduction of the intracellular K^+^ level in a relatively short time. Conversely, a reduction of anion component should reduce the K^+^ efflux due to the electrocoupling. For 1 mm thick root, an average K^+^ efflux of 100 nmol m^−2^ s^−1^ will lead to a reduction in the intracellular K^+^ concentration by 1 mM each 40 min, i.e., K^+^ loss of about 5 mM will be expected for the experiment presented in Figure 1B. Thus, providing that K^+^ leak is sustained, in just a few hours the intracellular K^+^ may reach a critically low level, which may switch the cell metabolism to a hibernation or even resulted in a programmed death scenario [7,24]. In barley roots, HO•-induced K^+^ efflux may transiently reach the values of 1500 and 10,000 nmol m^−2^ s^−1^ in mature and elongation root zones, respectively [10]. A higher efflux from the elongation zone is due to much poorer control of the membrane potential by the plasma membrane H^+^-ATPase and a higher functional expression of HO•-activated conductance; a simple calculation as above yields a decrease of intracellular K^+^ concentration by >20 mM in just 15 min in this case. Such a quick and massive loss of the intracellular solute, K^+^ and anions, will significantly reduce the turgor and would eventually affect the cell metabolic activity, as mentioned above.

Recently, Makavitskaya and co-workers [25] demonstrated that HO• production and related Ca^2+^ influx in the salinized roots is fuelled by ascorbate (Asc^−^) efflux. This is because Asc^−^ provided a reducing power for the transition metal ions, copper and iron, which in their reduced form are indispensable catalysers of Fenton reaction, the main source of the HO• generation in plants. The respective anion current was impermeable to large anions (gluconate), rapidly activated (reminiscent of the kinetics of anion currents mediated by Aluminium-activated Malate Transporters, ALMT) and weakly voltage-sensitive. This description shares similarities the properties of currents, recorded in the present study (Figure 3). Moreover, a rather low conductance of the HO•-activated current (Figure 2) poises its turnover at a margin between operation rates for the typical ion channels and ion transporters. Quite notably, Asc^−^ efflux from roots is ROS-activated [26]. Thus, the role of ROS (here, HO•)—induced anion current may go beyond a counterbalance of the K^+^ efflux, but to provide a positive feedback for the apoplastic HO• production, assuming that it is permeable for Asc^−^ It was shown previously that early HO•-induced conductance, ROSIC, is permeable to small anions (Cl^−^), but impermeable to HEPES [19]. Additional experiments are required to test whether this conductance may carry smaller naturally occurring organic anions, like ascorbate, malate or citrate.

In conclusion, by applying conventional intracellular microelectrodes for membrane potential monitoring, non-invasive ion fluxes measurements by the MIFE technique and whole-cell current recordings by patch-clamp, we provided additional experimental evidence for a dual, anion and cation, permeability of HO•-activated conductance across the plasma membrane. Such dual characteristics likely underlie a massive electrolyte loss, routinely observed under oxidative stress. The plausible experimental strategies for future studies should involve the MIFE technique for in vivo recordings of the HO•-induced anion fluxes and a detailed selectivity and pharmacological analysis of the ROSIC by the patch-clamp in the whole-cell mode.

## 4. Materials and Methods

### 4.1. Plant Material

Barley seeds (*Hordeum vulgare* v. “Gairdner”) were obtained from the Australian Winter Cereals Collection at TIA. Seeds were surface sterilized with 1% *v*/*v* HClO for 10 min, rinsed thoroughly with distilled water and were grown hydroponically in the dark at room temperature (23–25 °C) in aerated BSM solution, containing 0.5 mM KCl, 0.1 mM CaCl_2_. Seedlings with a root length of 60–80 mm were used for electrophysiological experiments. Seeds of pea (*Pisum sativum* “Greenfeast”) were external sterilized (commercial bleach for 30 min) and fully rinsed with distilled water. Seeds were germinated in a dark growth cabinet at +24 °C in two layers of wet paper in Petri dishes for 2 to 3 d. Homogeneously germinated seedlings were chosen for culture in a bubbled hydroponic unit (3-L plastic container). Seedlings were suspended on a plastic grid so that their roots were almost completely immersed in the growth solution. Aeration was provided by one aquarium air pump via flexible plastic tubing. Seedlings were grown under constant (+24 °C) conditions in a lighted growth cabinet until 5 d old. Roots of 8 to 10 cm long were used.

### 4.2. Membrane Potential Measurements

Conventional microelectrode (Harvard Apparatus Ltd., Kent, UK) with a tip diameter of ∼0.5 µm was filled with 1 M KCl and connected to a MIFE electrometer [14] via an Ag-AgCl half-cell. Pea seedlings were immobilized in the measuring chambers as described elsewhere [27] and preconditioned in BSM solution for 1 h. The mounted electrode was then impaled into the external cortex cells in the mature zone (20 mm from the tip) of intact roots using a manually operated hydraulic micromanipulator (MHW-4; Narishige) under microscopic control. MP values were monitored on a computer screen and recorded by the MIFE CHART software. All the treatments were administered after at least 3 min of stable low-noise recordings.

### 4.3. Non-Invasive Ion Flux (MIFE) Measurements

The intact roots of pea seeding were immobilized and preconditioned in BSM solution for an hour. Then the roots are exposed to HO• radical either by mixing 1 mM each of copper (CuCl_2_) and Na-ascorbate (as advised in previous studies [17,18,19]) or by adding 0.5 mM iron (as FeSO_4_) then 5 mM H_2_O_2_. The resulting transient H^+^, K^+^ and Ca^2+^ fluxes at the root mature zone were measured using Microelectrode Ion Flux Estimation technique (MIFE^TM^; University of Tasmania, Hobart, Australia). The basic principles of the MIFE^TM^ measurements and the specific details pertinent to microelectrode fabrication, calibration and measurements are available elsewhere [14]. The ion-selective resins used in preparing microelectrodes were calcium ionophore I-Cocktail A (Fluka # 21048), potassium ionophore I-Cocktail A (Fluka # 60031) and hydrogen ionophore II-Cocktail A (Fluka # 95297). To study the effects of cation (100 µM Gd^3+^ and 100 µM nifedipine) and anion-specific [100 µM NPPB—5-nitro-2-(3-phenylpropyl-amino) benzoic acid] blockers on HO• radical-induced fluxes, 1 mM Cu/Asc mixture was applied to the roots first, once the resulting transient Ca^2+^ fluxes switched from efflux to influx aforementioned blockers were added and the H^+^, K^+^ and Ca^2+^ fluxes measured continuously.

### 4.4. Patch-Clamp Measurements on Root Protoplasts

Epidermal protoplasts were isolated from pea root mature and elongation zones, >15 mm from the tip and 5–10 mm from the tip, respectively, as described previously [10]. Briefly, cell walls of root segments were digested by enzyme solution containing 2% (*w*/*v*) cellulase (Yakult Honsha, Tokyo, Japan), 1.2% (*w*/*v*) cellulysin (EMD Biosciences, San Diego, CA, USA), 0.1% (*w*/*v*) pectolyase, 0.1% (*w*/*v*) bovine serum albumin, 10 mM KCl, 10 mM CaCl_2_, and 2 mM MgCl_2_, pH 5.7, adjusted with 2 mM MES, with osmolality set hypertonic (780 mOsm) by sorbitol. After a 30-min incubation of root segments at 30 °C on a 90-rpm rotary shaker, the preparation was rinsed with the same solution without enzymes. To release protoplasts, the solution in preparation chamber was exchanged to a hypotonic (380 mOsm) one, containing 10 mM KCl, 2 mM CaCl_2_, and 1 mM MgCl_2_, pH 5.7. After removing the root debris, released protoplasts were washed by a solution applied for patch-clamp assays Measurements were made by means of an Axopatch 200A patch-clamp amplifier (Axon Instruments, Union City, CA, USA). Patch pipettes were pulled in several steps on a Flaming-Brown P-97 micropipette puller (Sutter Instruments, Novato, CA, USA) and fire polished on an L/M CPZ- 101 microforge (List-Medical) immediately before the experiment. The pipette solution contained (in mM) 100 KOH-HEPES (pH 7.4), 3 MgCl_2_, 0.8 CaCl_2_, and 2 K_2_EGTA; bath solution contained (in mM) 5 KCl, 2 CaCl_2_, 0.5 MgCl_2_, and 2 MES-KOH (pH 6). All patch solutions were adjusted to 550 mOsm by sorbitol. The patch pipette resistance with this solution combination was 5–10 MΩ. To ensure easy high-resistance (>5 GΩ) recordings, fresh protoplasts were used shortly (<30 min) after their release. Once the stable whole-cell recording was established for approximately 15 min, a 0.3 mM CuCl_2_/ 1 mM Na-ascorbate mixture was added to the bath to generate HO•. Ion currents were evoked by a sequence of rectangular voltage pulses from −160 to +100 mV in 20 mV increments. Records were taken every 2 min. For noise analysis steady-state currents were recorded over 5 s at fixed potentials (−100 or +60 mV) at different time points. The variance (σ^2^) was calculated by subtracting mean current from the free-running current value at every time point; mean square of resulting values was calculated for each sample for two fixed voltages, −100 and +60 mV. This was repeated for three individual protoplasts and the data were plotted as mean ± SE against the mean current value (Figure 2). After 40 min^−1^ h of stable recording, the bath solution was exchanged to the same one plus extra 80 mM NaCl. The reversal potential values for the whole-cell current before and after the solution exchange were evaluated and plotted separately for each individual protoplast (Figure 3B).
